# Enhanced myometrial vascularity: tailored superselective flow-reduction uterine artery embolization for fertility preservation

**DOI:** 10.1186/s42155-026-00679-7

**Published:** 2026-04-20

**Authors:** Vinicius Adami Vayego Fornazari, Gloria Maria Salazar, Gustavo Henrique Vieira Andrade, Thiago Franchi Nunes, Joaquim Mauricio Motta-Leal-Filho, Renato Abu Hana, Dherraj Reddy Goppireddy, Grit Armstrong Adler

**Affiliations:** 1 Division of Vascular and Interventional Radiology, Department of Radiology, University of Florida, College of Medicine – Jacksonville, Jacksonville, FL 32209 USA; 2 Division of Vascular Interventional Radiology, Department of Radiology, University of North Carolina, Chapel Hill, NC USA; 3 University of Iowa Carver College - Medicine University of Iowa, Iowa City, IA USA; 4 Interventional Radiology – Interventix, Campo Grande, MS Brazil; 5 Departamento de Radiologia, Faculdade de Medicina, Universidade de São Paulo, São Paulo, Brazil

**Keywords:** Enhanced myometrial vascularity, Uterine artery embolization, Superselective, Embolization, Fertility preservation, Reproductive-age women, Abnormal uterine, Bleeding

## Abstract

Enhanced myometrial vascularity (EMV) is a rare, pregnancy-related hypervascular condition that can cause persistent uterine bleeding and is frequently misinterpreted as an arteriovenous malformation. Women undergoing assisted reproduction who developed symptomatic EMV after miscarriage or uterine instrumentation. Superselective flow-reduction uterine artery embolization (UAE) was performed using 2.0–2.4 F microcatheters with individualized embolic selection, microspheres for diffuse thin-walled vessels and coils or NBCA for discrete feeders. The goal was targeted flow reduction while preserving global uterine perfusion. All patients experienced complete bleeding resolution, preserved uterine morphology, and subsequently achieved successful conception through assisted reproduction. These findings support tailored, fertilitypreserving UAE as a safe and effective management strategy for symptomatic EMV.

## Highlights

- Enhanced myometrial vascularity (EMV) is frequently misclassified as AVM, leading to unnecessary or excessive treatment.
- Superselective flow-reduction uterine artery embolization preserves fertility while treating EMV effectively.
- Tailoring embolic agents to lesion characteristics allows effective bleeding control without compromising uterine perfusion.
- All three patients resumed assisted reproduction and achieved successful pregnancies after treatment.
- This technique may serve as a first-line fertility-preserving intervention in reproductive-age women with EMV.

Dear Editor,

Enhanced myometrial vascularity (EMV) refers to an uncommon but clinically meaningful condition characterized by focal, pregnancy-related hypervascular myometrial pattern without true arteriovenous shunting, increasingly encountered by interventional radiologists in the post-partum or post-instrumentation setting. Unlike true arteriovenous malformations (AVMs), EMV does not exhibit complex arteriovenous shunting and is instead a form of marked hyperemia or neoangiogenesis. Clinically, it ranges from asymptomatic findings to severe, life-threatening bleeding [1–3]. Distinguishing EMV from AVM is critical to avoid overtreatment and unnecessary hysterectomy [3].

**Fig. 1:**
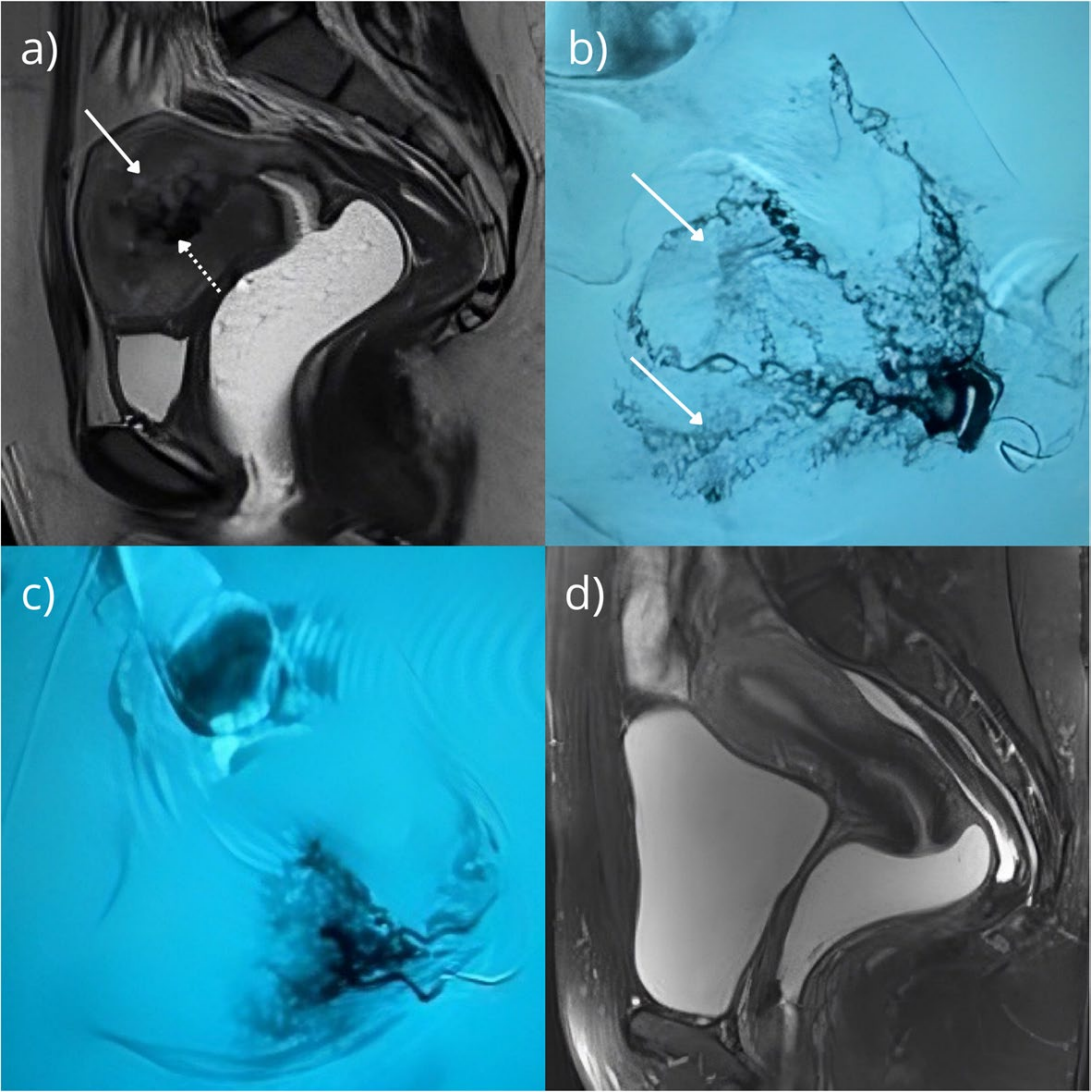
**a** Sagittal T2-weighted MRI demonstrating serpiginous flow-related signal voids (dashed arrow) with associated T2-hyperintense vascular lakes (solid arrow). **b** Left uterine artery angiogram shows dilated and spiraling intrauterine branches with apparent disorganization persistent in the uterine left lateral wall, in addition to blush areas (solid arrow); no nidus detected. **c** Left uterine artery angiogram post-embolization of 500–700 µm microspheres. Note the other intrauterine branches that were initially less opacified and later without filling opacification. **d** T2 Fat Sat 6 months after UAE shows no evidence of residual lesion and preservation of uterine morphology (myometrial and endometrial)

Diagnosis relies primarily on Doppler ultrasound (DUS), which demonstrates serpiginous anechoic structures with high-velocity, turbulent flow; a peak systolic velocity ≥ 0.83 m/s suggests elevated bleeding risk [1–3]. Magnetic resonance imaging (MRI) may reveal myometrial thickening and abnormal contrast enhancement (Fig. 1a). Uterine angiography (UA) remains the gold standard, typically showing focal and segmental hypervascularization with tortuous, disorganized spiral arteries, sometimes with a vascular lake-like appearance and early venous drainage (Fig. 1b) [3–5].

Management depends on symptom severity and reproductive plans. While mild cases may resolve spontaneously, persistent bleeding may require hormonal suppression, uterotonics, or surgical intervention. While hysterectomy can be offered to those not seeking future fertility [4, 5], UAE remains a minimally invasive option in all patients and does not preclude hysterectomy if unsuccessful. However, optimal management strategies, particularly in women undergoing assisted reproduction, remain poorly defined.

We report three reproductive-age women undergoing assisted reproduction who developed symptomatic EMV after miscarriage and manual vacuum aspiration (MVA). All presented with persistent abnormal uterine bleeding refractory to conservative therapy and expressed a strong desire for fertility preservation.

## Case series summary

- Patient 1: 37 years old, monochorionic pregnancy via embryo transfer, complicated by feto-fetal transfusion syndrome and placental retention. EMV diagnosed after significant post-MVA bleeding.
- Patient 2: 39 years old, euploid embryo transfer followed by embryonic pregnancy, spontaneous abortion, and EMV suspicion on imaging.
- Patient 3: 33 years old, in vitro fertilization with frozen blastocysts, spontaneous pregnancies complicated by miscarriages and EMV after repeated MVA. EMV suggested after radiology assessment.

UAE was performed via transfemoral 5 French access under local anesthesia and mild sedation, angiogram confirmed EMV without arteriovenous shunting. Superselective uterine artery embolization was performed by 2.0–2.4 F microcatheters to target lesion-specific feeding branches. Embolic agent selection followed established principles used in the management of post-partum hemorrhage. In cases with diffuse hypervascularity without a focal bleeding point, non-permanent embolic agents such as Gelfoam or microsphere (500–700 µm) were preferred to achieve flow reduction while preserving tissue perfusion. When a discrete focal feeder or pseudoaneurysm-like pattern was identified, coils or liquid embolic agents were selectively used. In the absence of arteriovenous shunting, complete devascularization was intentionally avoided to preserve uterine and ovarian perfusion (Fig. 1c).

All three patients experienced complete bleeding resolution and resumed assisted reproduction within 6 months. All subsequently achieved successful conception, resulting in ongoing pregnancies or full-term live births.

This experience highlights three technical concepts:

1. Superselective embolization—target only EMV-feeding branches to minimize ischemic injury to adjacent myometrium and endometrium, reducing non-target ovarian embolization risk.
2. Tailored embolic selection—prefer microspheres to balance flow reduction with tissue preservation; use coils or adhesives selectively.
3. Flow-reduction strategy—reduce inflow to the lesion while maintaining overall uterine perfusion, avoiding unnecessary bilateral embolization as in fibroid UAE.

Our results support tailored superselective flow-reduction UAE as a safe, effective, and fertility-preserving treatment for EMV. Larger studies are warranted to further define the role of this technique as a first-line therapy for symptomatic EMV in women of reproductive age.

## Data Availability

All imaging and clinical information were retrospectively retrieved from the institutional PACS along with clinical observations acquired.
